# Assessment of dietary pattern and nutritional status of undergraduate students in a private university in southern Nigeria

**DOI:** 10.1002/fsn3.759

**Published:** 2018-08-22

**Authors:** Kingsley Omage, Vivian O. Omuemu

**Affiliations:** ^1^ Department of Biochemistry College of Basic Medical Sciences Igbinedion University Okada Edo State Nigeria; ^2^ Department of Community Health College of Medical Sciences University of Benin Benin Edo State Nigeria

**Keywords:** dietary diversification, dietary pattern, nutritional status, undergraduates

## Abstract

**Background:**

Analysis of dietary patterns gives a more comprehensive impression of the food consumption habits within a population. Poor dietary habits among undergraduate students have been reported as a lifestyle challenge they face while in school. This study was carried out to assess the dietary pattern and nutritional status of undergraduate students in Igbinedion University, Okada.

**Methodology:**

This study applied a cross‐sectional, descriptive study design and 800 undergraduate students selected by multistage sampling method participated in the study. Data were collected using pretested, structured self‐administered questionnaires and anthropometric measurements were obtained. Data were analyzed using SPSS statistical package (version 22.0) and level of significance was set at *p* < 0.05.

**Results:**

Mean age of respondents was 23.5 ± 2.4 years, with a higher proportion being females (468; 58.5%). Over half of the respondents 448 (56.0%) skipped breakfast and 608 (76.0%) ate in between meals. More females 280 (59.8%) compared to males 168 (50.6%) skipped breakfast and the association between gender of respondents and breakfast skipping was statistically significant (*p* < 0.010). Majority of the respondents 744 (93.0%) ate snacks and the association between age group and snacking status of respondents was statistically significant (*p* < 0.034). Three hundred and ninety‐two (49.0%) of the respondents had high dietary diversity score while 212 (26.5%) had low dietary diversity score. The association between age group and dietary diversity was statistically significant (*p* < 0.001). More males 172 (51.8%) had a significantly (*p* < 0.004) higher dietary diversity score compared to the females 220 (47.0%). Over two‐thirds of the respondents 564 (70.5%) had normal BMI, 112 (14.0%) were overweight, and 76 (9.5%) were underweight.

**Conclusion:**

Skipping of breakfast and eating in‐between meals are common among the study population. Regular nutrition education program by the institution with emphasis on adequate dietary practices is recommended.

## 1 INTRODUCTION

Dietary pattern (DP) is the general profile of food and nutrient consumption which is characterized on the basis of the usual eating habits. The analysis of dietary patterns gives a more comprehensive impression of the food consumption habits within a population. It may be better at predicting the risk of diseases than the analysis of isolated nutrients or foods because the joint effect of various nutrients involved would be better identified (Hu, 2002). Also, since nutrient intakes are often associated with certain dietary patterns (Kant, Schatzkin, Block, Ziegler, & Nestle, 1991; Randall, Marshall, Graham, & Brasure, 1990) single‐nutrient analysis may be confounded by the effect of dietary patterns (Ursin et al., 1993). Patterns of nutritional behaviors adopted in childhood and adolescents are mostly continued in adult life and increase the risk of development of many chronic diseases (Kapka‐Skrzypczak et al., 2012). Diets in childhood and adolescents have public health implications due to evidence relating poor nutrition in childhood to subsequent obesity and elevated risks for type 2 diabetes, metabolic syndrome, and cardiovascular diseases (Canete, Gil‐Campos, Aguilera, & Gil, 2007), which are increasing in prevalence (WHO, 2004).

Nutritional status is the sum total of an individual's anthropometric indices as influenced by intake and utilization of nutrients, which is determined from information obtained by physical, biochemical, and dietary studies (Durnin & Fidanza, 1985). It is a result of interrelated factors influenced by quality and quantity of food consumed and the physical health of the individual. An adolescents’ nutritional status has important implications for his health, development of several chronic diseases, and plays a key role in breaking the cycle of malnutrition. The transition from adolescence to adulthood is an important period for establishing behavioral patterns that affect long‐term health and chronic disease risk (Meg, Small, Bailey‐Davis, & Maggs, 2012). University students seem to be the most affected by this nutritional transition (Baldini, Pasqui, Bordoni, & Maranesi, 2009; Wickramasinghe et al., 2004). Studies have shown that adolescence leaving their parents and living away from home to attend college experience numerous health‐related behavioral changes, which includes adoption of unhealthy dietary habits (Cluskey & Grobe, 2009; Strong, Parks, Anderson, Winett, & Davy, 2008; Wengreen & Moncur, 2009). These adopted habits are mostly attributed to drastic changes in the environment and resources available, frequent exposure to unhealthy foods and habits (Huang et al., 2003).

Many undergraduate students are adolescents who encounter numerous health risks along the path to adulthood, many of which affect quality of life and life expectancy. Studies have shown that youths are particularly vulnerable to poor eating habits and are said to be in the habit of eating “junks” (Papadaki & Scott, 2002). These poor eating habits may likely arise from lack of knowledge of the cumulative effects of their eating habits. In Nigeria, where there is an increase in fast food centers in its urban cities, it is a major concern (Ajala, 2006; Akinwusi & Ogundele, 2005). Most undergraduates are likely to be responsible for their diets for the first time away from home, therefore they need guidance on how to make informed dietary choices (Satia, Galanko, & Siega‐Riz, 2004). Other studies have linked the lifestyle of students, especially breakfast consumption, to their mental abilities which is reflected in their academic performance (Lisa, 1998; Pollit, Watkins, & Husaini, 1997). However, most of these studies have excluded young adults in the tertiary institution. Since poor dietary habits is a lifestyle challenge undergraduate students face while in school, this study was therefore carried out to assess the dietary pattern and nutritional status of undergraduate students in Igbinedion University, Okada, Edo State.

## 2 METHODOLOGY

### 2.1 Study design

The study applied a cross‐sectional, descriptive study design.

### 2.2 Study area

The study was carried out in Igbinedion University, Okada, located in Ovia North East Local Government Area of Edo State, Nigeria. It is an inland state in southern Nigeria which shares boundaries with three other states of the federation. Ovia North‐East, one of the 18 Local Government Areas in the State, has its headquarters in the town of Okada where Igbinedion University is located. It has an area of 2,301 km² and a population of 153,849 at the 2006 census. All the students are accommodated in the University's hostels with facilities for cooking and canteens for those who prefer to eat out.

### 2.3 Study population

The study population included undergraduate students of Igbinedion University Okada.

#### 2.3.1 Inclusion criteria

The full time undergraduate students attending the University who agreed to participate in the study and signed the consent form.

#### 2.3.2 Exclusion criteria

The undergraduate students excluded from the study were those who were pregnant (for females), on any form of medication, acutely ill or with known chronic diseases.

### 2.4 Sample size determination

Sample size (*n* = 800) was determined using the Fischer formula (*n* = *z* ^2^ *pq*/*d* ^2^) (Fisher, Laing, Stoeckel, & Townsend, 1991) as the population from which the sample size was drawn was more than 3,000.

### 2.5 Sampling technique

The study applied a multistage sampling method.

#### 2.5.1 Stage 1: Selection of faculties

Four (4) faculties were randomly selected from the seven (7) faculties in the school, using simple random sampling technique.

#### 2.5.2 Stage 2: Selection of departments

Two (2) departments each were randomly selected from the selected faculties using simple random sampling technique, giving a total of eight (8) departments.

#### 2.5.3 Stage 3: Selection of respondents

One hundred (100) respondents (undergraduate students) were selected from each selected department by simple random sampling method.

### 2.6 Data collection method

Data were collected using a structured dietary pattern questionnaire. Information on socio demographic characteristic, feeding habit, and dietary practices were obtained using self‐administered questionnaires to respondents. Each questionnaire was coded with a unique number representing each respondent. The questionnaire was used to collect the following information;

#### 2.6.1 Socio‐demographic characteristics

This comprise information on age, gender, course of study, year of study and this provided background information of students recruited into the study.

#### 2.6.2 Dietary practices assessment or feeding habits

Data were collected on the number of meals consumed daily, meal patterns, snacking habits, source of meals taken while in school, alcohol intake, and weekly food frequency consumption of nine food groups among the 8–12 recommended (FAO/WHO, 2003). The evaluation for the number of meals consumed in a day was based on recommendation by World Health Organization (FAO/WHO, 2003).

#### 2.6.3 Anthropometric measurements

Anthropometric measurements were taken with the respondents wearing light clothes and no shoes.

##### Weight measurement

To ensure reliable measurements of body weight using the mechanical bathroom scale, the scale was zeroed before the respondent stepped onto it. The respondents were asked to remove any ‘heavy’ items from their pockets (key's, wallets etc.) and remove any heavy items of clothing or apparel (big jackets, shoes, woollen jerseys etc.). They were asked to look straight ahead and stay still on the scales. The needle/digital screen was allowed to settle before the measurement was recorded. The body weight (kg) was measured to the nearest 0.5 kg. (Ambrosini et al., 2009).

##### Height measurement

Height measurement was taken using a “drop down” tape measure fixed at about 2 m on a wall. The respondents were asked to remove their shoes prior to taking the measurement. They were asked to stand with their back to the wall and look directly forward. The back of their feet, calves, bottom, upper back, and the back of their head should be in contact with the wall. They were positioned directly underneath the drop down measuring device. The measuring device was lowered until it rested gently on the top of the respondent's head and the measurement was recorded. Their height (m) to the nearest 0.5 cm was recorded (Ambrosini et al., 2009).

#### 2.6.4 Pretesting of questionnaires

The questionnaire was pretested by administering it to 10% of undergraduate students (80) in the University of Benin. The choice of undergraduate students of the University of Benin was based on the similarities in the characteristics of the undergraduate students (respondents) of the University of Benin to the undergraduate students (respondents) of Igbinedion University, Okada.

BMI (body mass index); was calculated as the weight in kilograms divided by the square of the height in meters. It is classified as <18.5 (underweight), 18.5–24.99 (normal), 25.0–29.99 (overweight), 30.0–34.99 (obesity Class I), 35.0–39.99 (obesity Class II), > 40 (obesity Class III) (Strong et al., 2008).

#### 2.6.5 Food groups used in the dietary diversity score

Twelve food groups were used for the dietary diversity score (DDS). The food groups were namely; cereals, roots and tubers, vegetables, fruits, meat, eggs, fish/sea food, legumes/nuts and seeds, milk and milk products, oils and fats, condiments, and soft drinks. The DDS (a factor which indicates the different food groups and varieties consumed) was classified as low DDS (≤3 food groups), medium DDS (4–5 food groups), and high DDS (≥6 food groups). (FAO/WHO, 2003).This was calculated based on the number of food groups consumed by the correspondents within the study period.

### 2.7 Data analysis

Data analysis was performed using SPSS statistical package. The data collected was also analyzed using simple description analysis such as percentages and frequency counts. Statistical significance was set at *p* < 0.05.

### 2.8 Ethical consideration

Ethical clearance for this study was obtained from the Ethics and Research Committee, University of Benin Teaching Hospital (UBTH). Permission was obtained from the school management and informed consent sought from the respondents.

**Protocol Number: ADM/E 22/A/VOL. VII/14519.**

**Date: 23 August 2017.**

## 3 RESULTS

The dietary pattern and Nutritional status of undergraduate students in Igbinedion University, Okada, Edo State Nigeria, were assessed using self‐administered questionnaires. Eight hundred (800) undergraduate students (respondents), both male (*n* = 332; 41.5%) and female (*n* = 468; 58.5%), participated in the study. Information on sociodemographic characteristics, dietary practices or feeding habits and anthropometric measurements were obtained.

The mean age of respondents was 23.5 ± 2.4 years with a higher proportion 328 (41.0%) seen to be in the 19–21 year age group. The least proportion of respondents 36 (4.5%) belonged to the ≥28 years age group. Over half of the respondents 468 (58.5%) were females and 788 (98.5%) were single. Majority of the respondents 884 (85.5%) were Christians and 164 (20.5%) belonged to the Igbo ethnic group. A higher proportion of the respondents 441 (55.1%) had greater than three siblings and eight (1.0%) had no siblings (Table 1).

**Table 1:** Sociodemographic characteristics of respondents

Variables	Frequency (*n* = 800)	Percent
Age group (years)		
16–18	120	15.0
19–21	328	41.0
22–24	240	30.0
25–27	76	9.5
≥28	36	4.5
Mean age ± *SD* (years)	23.5 ± 2.4	
Gender		
Male	332	41.5
Female	468	58.5
Marital status		
Single	788	98.5
Married	12	1.5
Religion		
Christianity	684	85.5
Islam	112	14.0
ATR	4	0.5
Ethnic group		
Igbo	164	20.5
Bini	148	18.5
Hausa	112	14.0
Yoruba	100	12.5
Esan	76	9.5
Urhobo	52	6.5
Ijaw	52	6.5
Etsako	40	5.0
Others^a^	54	7.0
Number of siblings		
None	8	1.0
1	52	6.5
2	149	18.6
3	150	18.8
>3	441	55.1

a Nembe, Tiv, Itsekiri, Owan, Isoko.

Three hundred and fifty‐six (44.5%) of the respondents ate two main meals in a day and 28 (3.5%) age more than three main meals in a day. The usual source of food for a higher proportion of the respondents 460 (57.5%) was both prepared and purchased. Over half of the respondents 448 (56.0%) skipped breakfast and 608 (76.0%) ate in between meals. Majority of the respondents 744 (93.0%) ate snacks, 556 (69.5%) and 760 (95.0%) did not take alcohol and did not smoke tobacco respectively (Table 2).

**Table 2:** Meal pattern of respondents

Variables	Frequency (*n* = 800)	Percent
Number of main meals in a day		
1	80	10.0
2	356	44.5
3	336	42.0
>3	28	3.5
Usual source of food		
Prepared only	308	38.5
Purchased only	32	4.0
Both	460	57.5
Breakfast skipping		
Yes	448	56.0
No	352	44.0
Eats in between meals		
Yes	608	76.0
No	192	24.0
Eats snacks		
Yes	744	93.0
No	56	7.0
Alcohol intake		
Yes	244	30.5
No	556	69.5
Tobacco smoking		
Yes	40	5.0
No	760	95.0

In the week preceding the survey, 404 (50.5%) of the respondents took breakfast sometimes, 352 (44.0%) and 44 (5.5%) ate breakfast everyday and did not take breakfast respectively. Over half 404 (50.5%) took lunch everyday and 40 (5.0%) had no lunch. Two thirds of the respondents 532 (66.5%) ate dinner everyday and 12 (1.5%) did not eat dinner (Table 3).

**Table 3:** Frequency of intake of the main meals in the week preceding the survey

Variables	Frequency (*n* = 800)	Percent
Breakfast		
Everyday	352	44.0
Sometimes	404	50.5
None	44	5.5
Lunch		
Everyday	404	50.5
Sometimes	356	44.5
None	40	5.0
Dinner		
Everyday	532	66.5
Sometimes	256	32.0
None	12	1.5

In the week preceding the survey, two‐thirds of the respondents 528 (66.0%) and 432 (54.0%) ate cereals and foods belonging to the roots and tubers food group ≥3 times. Five hundred and twenty (65.0%) and 504 (63.0%) ate vegetables and eggs, respectively, <3 times. Over half of the respondents 412 (51.5%) and 468 (58.5%) ate fruits and meat, respectively, ≥3 times (Table 4).

**Table 4:** Frequency of intake of different food groups by respondents in the week preceding survey

Food groups	Frequency (*n* = 800)	
	<3 times Frequency (%)	≥3 times Frequency (%)
Cereals	272 (34.0)	528 (66.0)
Roots and tubers	368 (46.0)	432 (54.0)
Vegetables	520 (65.0)	280 (35.0)
Fruits	388 (48.5)	412 (51.5)
Meat	332 (41.5)	468 (58.5)
Eggs	504 (63.0)	296 (37.0)
Fish/sea food	524 (65.5)	276 (34.5)
Legumes, nuts and seeds	520 (65.0)	280 (35.0)
Milk and milk products	392 (49.0)	408 (51.0)
Oils and fats	136 (17.0)	664 (83.0)
Condiments	376 (47.0)	424 (53.0)
Soft drinks	404 (50.5)	396 (49.5)

**Figure 1:**
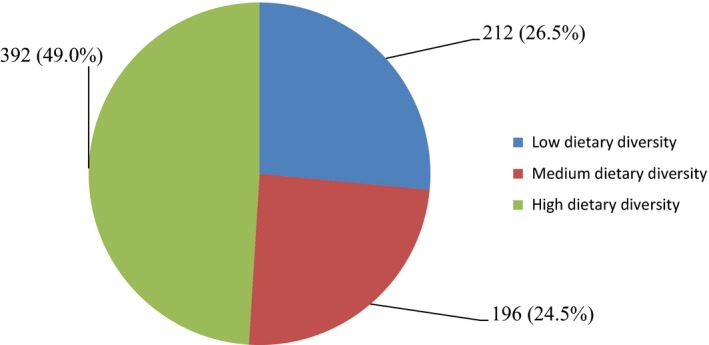
Overall dietary diversity score of respondents in the week preceding the survey

Three hundred and ninety‐two (49.0%) of the respondents had a high dietary diversity, 212 (26.5%) had a low dietary diversity and 196 (24.5%) had a medium dietary diversity (Figure 1).

**Figure 2:**
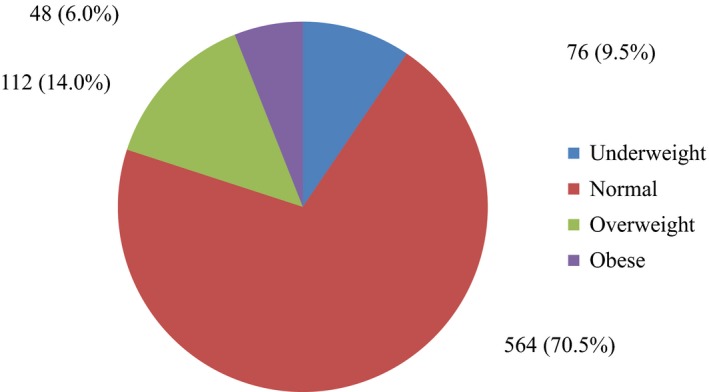
Body mass index classification of respondents

Over two‐thirds of the respondents 564 (70.5%) had a normal BMI, 112 (14.0%) were overweight and 76 (9.5%) were underweight (Figure 2).

Overweight and obese respondents were more among those who had a medium dietary diversity, 36 (18.4%) and 16 (8.2%), respectively, however, this finding was not statistically significant (*p* = 0.057) (Table 5).

**Table 5:** Overall dietary diversity by BMI classification of respondents

Variable	BMI classification (*n* = 800)				Test statistic	*p*‐Value
	Underweight (*n* = 76) Frequency (%)	Normal (*n* = 564) Frequency (%)	Overweight (*n* = 112) Frequency (%)	Obese (*n* = 48) Frequency (%)		
Overall dietary diversity						
Low	20 (9.4)	152 (71.7)	32 (15.1)	8 (3.8)	*χ* ^2^ = 12.248	0.057
Medium	12 (6.1)	132 (67.3)	36 (18.4)	16 (8.2)		
High	44 (11.2)	280 (71.4)	44 (11.2)	24 (6.1)		

## 4 DISCUSSION

Adolescence is one of the fastest growth periods of a person's life. During this time, physical changes affect the body's nutritional needs, while changes in one's lifestyle may affect eating habits and food choices. University students (undergraduates), within the age range of 17–30 years in every country constitute a large proportion of the total population. Many students do not know the nutritional values of the foods they eat. Some avoid certain foods because of personal dislike, social and cultural pressure, peer group influence, religion etc. There is a relationship between obesity and food intake and dietary patterns or feeding habits in adolescents and undergraduates. DP represent a general profile of food and nutrient consumption, characterized on the basis of the usual eating habits. Some dietary patterns appear quite common among adolescents, to mention a few: snacking, usually on energy‐dense foods; meal skipping, particularly breakfast, or irregular meals; wide use of fast food; and low consumption of fruits and vegetables. It has also been observed that most of the students lack adequate fund or divert their feeding money to other frivolities and so skip some meals. These unhealthy habits can lead to under‐nourishment or over‐nourishment with the resultant increase in the susceptibility of avoidable diseases.

The dietary pattern of the respondents shows that most of them ate two or three main meals a day which is necessary for good health. This is similar to findings from a study carried out among university students in South‐Eastern states of Nigeria (Achinihu, 2009). However, majority of them either skip breakfast or eat in‐between meals. Majority of the respondents sometimes skip breakfast while minority of them sometimes skip lunch or dinner. Skipping of meals is a very common practice among undergraduates (Hayda & Maria, 2007; Juan et al., 2013; Kurubaran et al., 2012; Moy et al., 2009). Although breakfast is very important for the health and well‐being of the body, students may find it difficult to take as they are always in a hurry to go for their classes. Some may deliberately skip breakfast because of the consciousness of their body weight and appearance. This is more common among females who are more conscious of their diet (Carmel & Camilleri, 2011). Majority of the respondents ate snacks in‐between meals, possibly to enable them cope with the energy needs of the body as they go about their normal academic activities. The pattern also shows a high intake of snacks among them, just as observed among the university students in the South‐Eastern states of Nigeria (Achinihu, 2009), but smoking and intake of alcohol was very low. Majority of the students do not take alcohol or smoke, however, those who take alcohol or smoke, do so occasionally or rarely. The knowledge of the health implications of alcohol consumption and smoking may be responsible for avoidance of such practices among the respondents. The dietary pattern assessments of the undergraduate students (respondents) also indicates that majority of the students mostly prepare their food. Those who purchase their food do so in the canteen, although they seem not to be satisfied eating out. Lunch is often eaten out in the canteen while breakfast and dinner were mainly prepared. Eating out in canteens is a common practice among undergraduate students (Achinihu, 2009; Hayda & Maria, 2007). This may be because more time is spent outside their halls of residence during lunch periods usually in classrooms.

The meal pattern of the respondents also shows that majority of them consumed more of foods belonging to the cereals, roots and tubers, fruits, meats, oils and fats groups and less of foods belonging to the vegetables and eggs group. This may affect the availability of the nutrients (i.e., minerals and proteins) inherent in these food groups, to the respondents. The DDS of the respondents shows that more of them ate more than six food groups, which makes available necessary nutrients for optimal health. The dietary diversity of the respondents may be a reflection of better knowledge of basic nutritional values of the different food groups. High dietary diversity includes food from many food groups (≥6) which makes available balanced nutrients for optimal health. The high DDS of the respondents indicates their consumption of wide varieties of food which ensures availability of useful and balanced nutrients. This guarantees optimal nutrition which has a positive impact on their nutritional and health status.

Most of the undergraduate students of Igbinedion University, Okada who participated in the study had normal BMI, and therefore seem to be well nourished. The proportion of overweight respondent increased despite meal skipping and avoidance of certain food groups. Prevalence of overweight and obesity among the females may result from excessive consumption of selected food groups which are mostly energy‐dense or refined and avoidance of food groups with low energy and necessary vitamins and minerals. This may be true as those who had medium dietary diversity were more obese and overweight than those who had high dietary diversity. This is dangerous for their health as obesity or overweight predisposes them to risk of NCDs (Van den Berg, Abera, Nel, & Walsh, 2013).

In conclusion, the study reveals that most of the respondents ate two or three main meals a day, either skip breakfast or eat in‐between meals. The high DDS of the respondents ensures balanced nutrition. BMI of the respondents indicate that majority of them had a normal BMI. However, the undergraduate students (respondents) of Igbinedion University, Okada need health education on the benefits of good dietary practices to achieve optimal health.

## CONFLICT OF INTEREST

The authors declare that they do not have any conflict of interest.

## ETHICAL STATEMENT

The study conforms to the Declaration of Helsinki, United States, and/or European Medicines Agency Guidelines for human subjects. This study's protocols and procedures were ethically reviewed and approved by the Institutional Review Board (Ethics and Research Committee) of the University of Benin Teaching Hospital (UBTH). *Informed Consent*: Written informed consent was obtained from all study participants.
